# Alcohol Use Among Adolescents and Young Adults

**Published:** 2003

**Authors:** Michael Windle

**Affiliations:** Michael Windle, Ph.D., is a professor of psychology and director of the Center for the Advancement of Youth Health, University of Alabama at Birmingham

**Keywords:** adolescent, young adult, high school student, undergraduate student, high-risk youth, AOD (alcohol and other drug) use, prevalence, epidemiological indicators, AOD associated consequences, risk-taking behavior, gender differences, United States

## Abstract

National surveys of adolescents, college students, and other young adults in the United States reveal high rates of alcohol use among these age groups as well as high rates of dangerous drinking practices such as binge drinking and daily drinking. Additional health-compromising behaviors such as tobacco use and drinking and driving often co-occur with alcohol use in these populations. The physical locations or drinking contexts where alcohol use occurs can predict drinking practices and consequences. This information can be used to identify appropriate targets for effective interventions and social policies.

Alcohol use during adolescence and young adulthood remains a prominent public health problem in the United States. National survey results indicate that 28.6 percent of 12th graders and 40.1 percent of college students reported binge drinking (i.e., consuming five or more drinks in a row) during the 2-week period preceding the survey (Johnston et al. 2003*a*,*b*). Alcohol use among adolescents and college students is also associated with a broad array of risk behaviors, including tobacco use and drinking and driving. In addition, studies on college campuses have shown that students who do not drink nevertheless experience adverse secondhand effects of drinking, including victimization (e.g., verbal or physical threats and actions) and personal intrusion (e.g., disruption of sleep or study habits) by those who have been drinking (Wechsler et al. 1998). Another disturbing trend in youth drinking is the initiation of alcohol use at younger ages. Between 1987 and 1996, surveys have shown that the average age of initiation to alcohol use decreased by more than 1.5 years, from 17.8 years in 1987 to 15.9 years in 1996 (Office of National Drug Control Policy 1997).

In 1999, more than 32 percent of young people reported beginning to drink before age 13 (Centers for Disease Control and Prevention [CDC] 2000). Earlier initiation of alcohol use (prior to age 15) has been associated with increased risk for alcohol-related problems later in life (Grant and Dawson 1997). This article reviews epidemiological data on alcohol use among adolescents, college students, and young adults not in college, and presents data on the physical locations or drinking contexts where alcohol use occurs. In addition, this article discusses the prevalence of health-compromising behaviors that often co-occur with youth drinking, such as smoking, illicit drug use, and risky sexual behaviors.

## Prevalence of Adolescent Alcohol Use

Findings from the Monitoring the Future Survey (MFS) (Johnston et al. 2003*a*)—for which a nationally representative sample of 8th, 10th, and 12th graders are surveyed each year on alcohol and other drug use—indicate a very high rate of alcohol use in this population. Table 1 presents data on two common indicators of adolescent alcohol use, the percentage of respondents who report having consumed alcohol in the 30 days before the survey (30-day prevalence) and the percentage of those who report having been drunk within the previous 30 days. There are clear trendstoward higher prevalence of both 30-day use and having been drunk in the last 30 days among those in higher grades; it is significant that 19.6 percent of 8th graders reported using alcohol in the previous 30 days, and 6.7 percent reported having been drunk during that time. All of these students are legally underage drinkers. Furthermore, the high prevalence of drinking at an early age bodes ill for psychosocial development among youth because of the increased risk for both alcohol-related problems (e.g., poorer school performance, more substance-using peers) and other co-occurring problems (e.g., delinquency, sexual activity) (Windle 1999). Consistent with national surveys conducted in previous years, geographically the Northeast and North Central regions had the highest prevalence of alcohol use by young people, followed by the West and South. There were few differences between metropolitan and nonmetropolitan areas. Among racial and ethnic groups, White youth generally had the highest prevalence of alcohol use, followed by Hispanics. African Americans had significantly lower levels of alcohol use compared with Whites or Hispanics across all grade levels (Johnston et al. 2003*a*).

### Gender Comparisons

Researchers have suggested that the differences in alcohol use between males and females are converging (White and Huselid 1997), but it appears that there is more convergence on some alcohol indicators than others. The data in table 1 indicate that male and female 8th and 10th graders had similar rates of both using alcohol and having been drunk in the previous 30 days. Nonetheless, gender differences emerge among 12th graders, with males having a higher 30-day prevalence of alcohol use and of having been drunk.

Table 2 presents data for males and females on additional indicators of alcohol use. These findings indicate an approximately equal prevalence of lifetime alcohol use for boys and girls at each grade level. By the 12th grade, however, marked gender differences emerge in binge drinking in the previous 2 weeks and in daily use of alcohol (defined as drinking alcohol on 20 or more occasions in the past 30 days). Hence, although alcohol use and dangerous drinking practices (e.g., getting drunk, binge drinking) occur among both boys and girls, these practices are more prevalent among boys by the 12th grade.

### Adolescents in Alternative Schools

Children attending alternative high schools constitute a distinctive subpopulation of adolescents who may be at increased risk for the development of serious alcohol problems. Alternative high schools serve students at risk for failing or dropping out of regular school and students who have been removed from their regular high schools because of behavior problems such as violence or substance use. About 2 percent (280,000) of all high school students attend alternative schools (Quality Education Data, Inc. 1997). Grunbaum and colleagues (2000) reported that in 12th grade, 73.5 percent of boys and 60.1 percent of girls selected from a nationally representative sample of alternative schools reported consuming alcohol in the previous 30 days, and 55.4 percent of boys and 42.9 percent of girls reported consuming five or more drinks in a row during the previous 30 days. These rates of alcohol use were considerably higher than those for youth from regular high schools, as were the rates of a range of other health-compromising behaviors such as drinking and driving, suicide attempts, unintended pregnancies, and sexually transmitted diseases. Although these rates of alcohol use and co-occurring problems among adolescents in alternative schools are high, these schools have typically not been the focus of targeted interventions.

## Prevalence of College Student and Young Adult Alcohol Use

Rates of drinking among college students and other young adults are also high. College students are often undergoing role transitions—such as moving away from the family home for the first time, residing with other students, and experiencing reduced adult supervision—that may increase the risk of alcohol use and abuse. College students also frequently reside in different physical and social environments and encounter new social and institutional factors (e.g., college parties, football weekends) that may foster heavy alcohol use.

Table 3 presents findings from the MFS followup surveys among college students and other (noncollege) young adults (Johnston et al. 2003*b*). These are referred to as followup surveys because the young adult samples (both college and noncollege) were selected from high school students who had previously participated in the MFS during their senior year in high school. The college student sample consisted of full-time students, 1 to 4 years post-high school (ages 18 to 23), enrolled in a 2- or 4-year college. The noncollege sample consisted of adults ages 19 to 28 who were not full-time students. The findings indicate very high rates of alcohol use and binge drinking as well as a fairly large number of daily drinkers among both college and noncollege young adults. College females, relative to noncollege females, had a higher prevalence of using alcohol in the previous 30 days and a higher rate of binge drinking. College males had higher rates of binge drinking, alcohol use in the previous 30 days, and daily alcohol use than did noncollege males. Gender comparisons within the college and noncollege samples indicated a pattern similar to that observed in secondary students: Compared with females, males reported higher rates of more serious manifestations of alcohol use (i.e., significantly higher prevalences of binge and daily drinking).

As noted previously, the MFS followup (Johnston et al. 2003*b*) is limited to high school graduates. To supplement these data, findings for full-time college students and other young adults from the 2000 National Household Survey on Drug Abuse (NHSDA) (Substance Abuse and Mental Health Services Administration 2001) are provided in table 4. The NHSDA is not restricted to high school graduates and therefore provides a more representative sample of young adults. The findings in table 4 indicate patterns similar to those reported for the MFS; college students, compared with noncollege young adults, have higher rates of previous 30-day alcohol use and binge drinking. These somewhat larger differences for the NHSDA sample than the MFS sample may be attributable to differences in the samples (i.e., the MFS sample consisted of high school graduates only). The measures of serious drinking used in the two surveys are not parallel, but the findings are similar. The NHSDA measured heavy episodic drinking, defined as having five or more drinks on the same occasion at least 5 days in the past 30 days, and found that heavy episodic drinking was higher among full-time college students. The MFS measured daily use of alcohol, and the prevalence was higher among college than noncollege young adults. Hence, both surveys indicated more serious drinking among college students compared with noncollege young adults.

Another study of college drinking, the 1995 National College Health Risk Behavior Survey (CDC 1997), investigated the prevalence of alcohol use and other risk behaviors among a national sample of 4,609 college students from 136 institutions. These findings, summarized in table 5, show gender differences similar to those reported in the other surveys: Males had higher prevalences than females of alcohol use during the previous 30 days, binge drinking during the previous 30 days, and daily drinking. Observed differences in alcohol use across racial/ethnic groups were also similar. African Americans reported the lowest prevalence of alcohol use and Whites the highest prevalence. The younger age group (18 to 24 years) had higher prevalences of drinking during the previous 30 days and binge drinking than did people 25 years and older. These age group differences may indicate that some older students are “maturing out” of heavier alcohol use or adopting adult social roles (e.g., spouse, parent) that are associated with decreased alcohol use (e.g., Miller-Tutzauer et al. 1991). However, it should be noted that the prevalence of daily drinking was higher among older respondents, which could reflect progression into dependent drinking by a portion of this population.

Both the prevalence of alcohol use during the last 30 days and the prevalence of binge drinking were higher among students at 4-year institutions than among students at 2-year institutions. The prevalence of daily drinking was slightly higher for women at 2-year institutions, compared with those at 4-year institutions, and was the same for men at the two types of institutions. Living conditions (e.g., dormitory rather than family home) and the increased role of fraternities and sororities on 4-year campuses compared with 2-year campuses may be important factors influencing the differences in rates of binge drinking and 30-day alcohol use at the two types of institutions.

As reviewed above, survey data provide a consistent picture of high rates of alcohol use, binge drinking, and daily drinking on college campuses. Another important index of alcohol use by college students is the extent of alcohol-related adverse consequences. Table 6 summarizes data on alcohol-related adverse consequences among college students, based on a nationwide survey of 54,444 undergraduates at 131 college campuses (Core Institute, Southern Illinois University 2001). These findings indicate that many students experience adverse consequences from drinking; 64.5 percent reported having had a hangover, 29.0 percent reported driving a car while under the influence, and 31.8 percent reported arguing or fighting. These high rates of adverse consequences are consistent with the high rates of alcohol use reported in the surveys cited above, and they do not portend well for the health and well-being of many college students. The influence of drinking context, such as place of residence for college students, is described below, along with findings on the secondhand consequences of college drinking.

## Drinking Contexts

Standard surveys about the prevalence of alcohol use generally do not gather information about key situational or contextual conditions, such as the location of drinking. This information, however, may have a significant bearing on the interpretation of survey findings and on the identification of appropriate targets for effective interventions and social policies.

Adolescents consume alcohol in a variety of contexts, including their own homes, homes of friends or acquaintances, bars or restaurants, outdoor settings (e.g., parks, beaches, sports stadiums), at school or work, and in moving cars or trucks. Based on a sample of 1,914 high school seniors, Lee and colleagues (1997) reported that the three most highly endorsed locations for drinking were in another person’s home, an outdoor setting, and a moving car or truck. Drinking outdoors and drinking in a car or truck were both significant predictors of alcohol-impaired driving (i.e., self-reported driving after drinking enough to affect driving), even when statistically controlling for several other predictor variables such as gender, discretionary income, number of drinking occasions in the last 30 days, and number of binge drinking episodes in the last 2 weeks. Drinking in a car or truck (including drinking by drivers) was associated with a 2.5 times higher probability of driving while impaired, and drinking outdoors was associated with a 1.5 times greater probability.

Several studies have indicated that living circumstances are a major contextual influence on college student drinking behaviors (Perkins 2002). For example, using data from the 1993 College Alcohol Study of 17,592 students from 140 campuses in 40 States and the District of Columbia, Wechsler and colleagues (1995) reported that fraternity residence (relative to non-fraternity residence) was associated with an almost sevenfold increase in the probability of bingeing or of episodic drinking. After statistically controlling for several other variables that might influence binge drinking in college—such as binge drinking in high school, school grades, cigarette use, and importance of religion—fraternity residence was still associated with a fourfold increase in the probability of binge drinking. Similar findings have been reported in other studies (Presley et al. 2002), though the presence of a Greek system does not necessarily correlate with a high percentage of binge drinkers among a college’s students. Fraternity residence could account for at least some of the college versus noncollege differences in episodic heavy drinking reported above. Similarly, with a representative sample of almost 2,000 New York State college students (ages 18 to 25 years), Barnes and colleagues (1992) reported that living in a dormitory instead of living at home with parents was associated with substantially higher levels of alcohol use and alcohol-related adverse consequences.

In another study of drinking contexts, Wechsler and colleagues (1998) used data from a 1997 College Alcohol Study that surveyed 14,521 students from 130 colleges. The researchers first categorized college campuses as low, medium, or high with respect to the prevalence of binge drinking among students. Binge drinking was defined as low on campuses if it was reported by less than 36 percent of students; medium, if reported by 36 to 50 percent of students; or high, if reported by more than 50 percent of students. The researchers then assessed the association between the level of binge drinking on campus and adverse secondhand effects caused by students who were drinking but experienced by students who were nonbingeing drinkers or abstainers. Secondhand effects included events such as having been insulted or humiliated; having been pushed, hit, or assaulted; having experienced an unwanted sexual advance; having had studying or sleep interrupted; and having had to take care of a drunken student. The findings indicated that significantly higher levels of adverse secondary effects occurred on campuses with higher levels of binge drinking. For example, 74 percent of nonbinge and abstaining students reported having had their studying or sleep interrupted on high binge-drinking campuses, compared with only 40 percent on low binge-drinking campuses. Similarly, 20 percent of students reported property damage on the high binge-drinking campuses, compared with only 6 percent on low binge-drinking campuses. Hence, binge drinking varies across campuses and can have negative effects for both the binge drinkers and nonbinge-drinking and abstaining students.

## Alcohol and Co-Occurring Health Risk Behaviors

Alcohol use among adolescents co-occurs with a range of other risky behaviors including tobacco use, sexual activity, violence, drinking and driving, and suicide (Windle 1999). Based on data collected from more than 4,000 adolescents (ages 13 to 18 years) in the 1995 NHSDA, Johnson and colleagues (2000) reported strong relationships between binge drinking and smoking. For example, adolescents who reported binge drinking in the 30 days before the survey were 17 times more likely to have smoked during that time than adolescents who did not report binge drinking. Also, among adolescents who did not binge drink in the previous 30 days, more than 82 percent did not smoke during that time. These findings indicate that alcohol use (in this instance, specifically binge drinking) and smoking are highly associated with each other.

Findings from the National College Health Risk Behavior Survey (CDC 1997) have also demonstrated significant associations between alcohol use and co-occurring risky behaviors among college students. For example, during the 30 days preceding the survey, 27.4 percent of college students nationwide reported driving a vehicle after consuming alcohol. Males were more likely to report driving after drinking than females (33.2 percent compared with 22.8 percent), and Whites (31.2 percent) and Hispanics (24.8 percent) were more likely than African Americans (14.7 percent) to report driving after drinking. Similarly, of the 76.8 percent of college students who had gone boating during the 12 months preceding the survey, 30.5 percent had consumed alcohol when boating or swimming. During the 30 days preceding the survey, 35.1 percent had ridden in a car with a driver who had been drinking alcohol. Among sexually active college students, 16.6 percent used alcohol or other drugs during their last sexual intercourse, and this rate was higher among younger students (19.3 percent among those ages 18 to 24) compared with older students (13.1 percent among those ages 25 years or older).

## Summary

Epidemiological findings on adolescent and young adult alcohol use reveal several disturbing trends. National data indicate not only that there are high rates of alcohol use among these age groups, but also that many adolescents and young adults engage in drinking practices (e.g., binge drinking, daily drinking) associated with major contributors to youth mortality (e.g., automobile crashes, suicide) and with disruptions in significant contexts (e.g., school, work, family) that are important for healthy development. The findings also indicate that there are important secondary effects (e.g., having been pushed, hit, or assaulted; having study habits or sleep disturbed) for abstainers and nonbinge drinkers on college campuses with high rates of binge drinking. The findings regarding gender differences indicate that almost as many females as males are consuming alcohol, but that males in adolescent, college, and noncollege samples have a higher prevalence than females of more serious drinking patterns such as binge drinking and daily drinking. Racial/ethnic group differences indicate the highest prevalence of use among Whites, with Hispanics second, and African Americans at substantially lower levels. These racial/ethnic group findings were consistent across the multiple probability samples referred to in this article.

In addition to the high rates of alcohol use among these youthful populations, survey data demonstrate the common co-occurrence of additional health-compromising behaviors (e.g., tobacco use, illicit drug use, risky sexual behavior) among heavier users. This information should be integrated in subsequent intervention efforts and social policies regarding the health and well-being of youth (Hingson and Howland 2002; Shulenberg and Maggs 2002). These findings also support concerted efforts to address the numerous problems associated with alcohol use among adolescents and young adults. In addition, the information provided in this article clearly suggests that there are distinct drinking subcultures on many college campuses that foster negative consequences for both drinkers and those around them. Interventions and social policies on college campuses must incorporate this information to effectively modify alcohol use and its adverse primary and secondary consequences on college campuses.

## Figures and Tables

**Table 1:** Prevalence of Having Used Alcohol, and Having Been Drunk in the 30 Days Preceding the Survey, Among Various Demographic Subgroups of 8th, 10th, and 12th Graders in 2002

	30-Day Prevalence of Alcohol Use (%)				30-Day Prevalence of Having Been Drunk (%)		
	
	Grade	8th	10th	12th	8th	10th	12th
**Total**		19.6	35.4	48.6	6.7	18.3	30.3
**Gende**r							
Male		19.1	35.3	52.3	7.1	19.3	34.3
Female		20.0	35.7	45.1	6.3	17.4	26.9
**Region of Country**							
Northeast		19.3	36.3	50.9	5.3	18.1	33.6
North Central		19.1	35.7	52.1	7.0	18.7	35.0
South		21.6	33.7	46.8	7.6	17.5	28.4
West		17.0	37.2	45.0	5.9	19.5	25.0
**Population Density**							
Large MSA*		17.4	32.0	50.3	5.2	16.5	32.9
Other MSA		20.1	35.2	48.8	7.3	18.0	29.1
Non-MSA		21.4	40.4	45.9	7.3	21.4	29.2
Race/Ethnicity**							
African American		14.8	24.3	30.1	4.0	8.6	12.1
White		21.5	40.0	54.0	8.0	23.2	36.6
Hispanic		26.5	37.9	47.5	8.4	17.4	23.5

* MSA = Metropolitan Statistical Area.

** To increase subgroup sample size and thereby provide more stable estimates, percentages were based on 2001 and 2002 data combined.

SOURCE: Johnston et al. 2003*a*.

**Table 2:** Prevalence of Ever Having Used Alcohol, Having Five or More Drinks in a Row in the 2 Weeks Preceding the Survey, and Daily Drinking* Among Gender Groups in 2002

	Grade	Ever Used Alcohol (%)			5+ Drinks in a Row in Last 2 Weeks (%)			Daily Use of Alcohol*		
		
		8th	10th	12th	8th	10th	12th	8th	10th	12th
Males		47.2	65.5	77.9	12.5	23.8	34.2	0.8	2.6	5.3
Females		46.8	68.5	78.5	12.1	21.0	23.0	0.4	1.0	1.7

* Daily use was defined as drinking alcohol on 20 or more occasions in the past 30 days.

SOURCE: Johnston et al. 2003*a*.

**Table 3:** Prevalence of Alcohol Use in the 30 Days Preceding the Survey, Having Five or More Drinks in a Row in the 2 Weeks Preceding the Survey, and Daily Drinking* Among College Students and Other Young Adults in 2000

	Used Alcohol in Previous 30 days (%)		5+ Drinks in a Row in Previous 2 Weeks (%)		Daily Use of Alcohol (%)	

	College Status					

	College	Noncollege	College	Noncollege	College	Noncollege
Males	70.2	65.5	50.7	43.8	7.0	5.3
Females	68.0	56.1	33.4	29.0	3.7	3.5
Total	68.9	60.1	40.1	35.4	5.0	4.3

* Daily use was defined as drinking alcohol on 20 or more occasions in the past 30 days.

NOTE: The table reflects findings from the Monitoring the Future followup surveys of college students and other (noncollege) young adults who had previously participated in the survey during their senior year in high school. The college student sample consisted of full-time students, 1 to 4 years post–high school, enrolled in a 2- or 4-year college. The noncollege sample consisted of adults ages 19 to 28 who were not full-time students.

SOURCE: Johnston et al. 2003*a*,*b*.

**Table 4:** Prevalence of Alcohol Use in the 30 Days Preceding the Survey, Having Five or More Drinks in a Row in the Past Month, and Heavy Episodic Drinking* in the Past Month, by College Students and Noncollege Young Adults Ages 18 to 22 in 2000

	30-Day Prevalence of Alcohol Use (%)	5+ Drinks in a Row, Last 30 Days (%)	Heavy Episodic Drinking (%)*
Full-Time College Students	62.0	41.4	16.4
Other Young Adults	50.8	35.9	12.1

* Heavy episodic drinking was defined as having five or more drinks on the same occasion at least 5 days in the past 30 days.

SOURCE: Substance Abuse and Mental Health Services Administration 2001.

**Table 5:** Prevalence of Alcohol Use in the 30 Days Preceding the Survey, Having Five or More Drinks in a Row in the Preceding 30 Days, and Daily Drinking* Among College Students, by Gender, in 1995

	30-Day Prevalence of Alcohol Use (%)		5+ Drinks in a Row Last 30 Days (%)		Daily Drinking (%)*	

	Female	Male	Female	Male	Female	Male
**Age Group**						
18–24 years	67.0	73.2	34.8	48.7	1.6	5.4
25 years and older	60.8	71.8	15.7	32.2	3.1	9.2
**Race/Ethnicity**						
African American	49.0	62.6	6.1	22.8	1.1	2.4
White	69.7	75.7	31.6	49.4	2.5	7.6
Hispanic	58.0	71.2	22.6	39.9	1.1	3.0
**Institution Type**						
2-year	59.4	67.9	20.6	34.8	2.5	6.6
4-year	69.0	76.6	32.8	50.7	1.9	6.6
**Tota**l	64.5	72.9	27.0	43.8	2.2	6.6

* Daily drinking was defined as drinking alcohol on 20 or more days in the 30 days preceding the survey.

SOURCE: Centers for Disease Control and Prevention 1997.

**Table 6:** Annual Prevalence of Adverse Consequences Associated With Alcohol Use Among College Students in 2001

Adverse Consequences	Percentage
Had a hangover	64.5
Got nauseated or vomited	55.5
Did something they later regretted	40.5
Missed a class	34.1
Drove a car while under the influence	29.0
Got into an argument or fight	31.8
Were criticized by someone they know	32.3
Performed poorly on a test/other project	24.4
Had trouble with police or other authorities	16.5

SOURCE: Core Institute 2001.
